# The self-fulfilling prophecy in medicine

**DOI:** 10.1007/s11017-024-09677-z

**Published:** 2024-08-09

**Authors:** Mayli Mertens

**Affiliations:** 1 Department of Philosophy, Center for Ethics, University of Antwerp, Antwerp, Belgium; 2 Department of Public Health, Center for Medical Science and Technology Studies, University of Copenhagen, Copenhagen, Denmark; 3 Atlas Bioethics Center, Andalusia, Spain

**Keywords:** Self-fulfilling prophecy, Reflexive prediction, Operative SFP, Transformative SFP, False positive, True positive, Research ethics

## Abstract

This article first describes the mechanism of any self-fulfilling prophecy through discussion of its four conditions: credibility, employment, employment sensitivity, and realization. Each condition is illustrated with examples specific to the medical context. The descriptive account ends with the definition of self-fulfilling prophecy and an expansion on collective self-fulfilling prophecies. Second, the normative account then discusses the moral relevance of self-fulfilling prophecies in medicine. A self-fulfilling prophecy is typically considered problematic when the prediction itself changes the predicted outcome to match the prediction (*transformative self-fulfillment*). I argue that also self-fulfilling prophecies that do not change the outcome but change the ways in which the outcome was realized (*operative self-fulfillment*), have significant ethical and epistemic ramifications. Because it is difficult to distinguish, retrospectively, between a transformative and an operative self-fulfilling prophecy, and thus between a false or true positive, it becomes equally difficult to catch mistakes. Moreover, since the prediction necessarily turns out true, there is never an error signal warning that a mistake might have been made. On the contrary, accuracy is seen as the standard for quality assurance. As such, self-fulfilling prophecies inhibit our ability to learn, inviting repetition and exacerbation of mistakes. With the rise of automated diagnostic and prognostic procedures and the increased use of machine learning and artificial intelligence for the development of predictive algorithms, attention to self-fulfilling feedback loops is especially warranted. This account of self-fulfilling prophecies is practically relevant for medical research and clinical practice. With it, researchers and practitioners can detect and analyze potential self-fulfilling mechanisms in any medical case and take responsibility for their ethical and epistemic implications.

## Introduction

A reflexive prediction is a prediction that impacts the eventual outcome or the way that the outcome was realized. Especially self-defeating and self-fulfilling prophecies (SFPs) have long fascinated scholars and practitioners in many fields. In medicine, the placebo effect is one of the most longstanding and commonly acknowledged SFPs [1, 2]. Although it became the standard quality assurance for trials in medical research, controversy remains about using the placebo for therapeutic purposes [3–7]. Because the placebo carries an optimistic prediction—namely that it will make the patient better—its self-fulfilling effect is positive, thereby improving the patient’s health. Pessimistic self-fulfilling prophecies, like the nocebo effect, have a negative impact. Pessimistic SFPs are particularly worrisome in prognostic and early-diagnostic practices as they can be the cause of the eventual poor outcome.

Ironically, and in contrast to this phenomenon, the very purpose of diagnostic and prognostic tests is to follow bad news (i.e. positive test results) with an action that has a *self-defeating* reflexive impact, not a self-fulfilling one. Take for instance a positive test for BRCA1 or 2 gene mutations. This gives the person who got tested reason to believe they have an increased chance of developing breast cancer. This credible test result is followed by a decision to get a double mastectomy, removing all breast tissue, effectively making it impossible for breast cancer to develop there. The prediction was thus given credibility and then employed, the risk of developing cancer was sensitive to the removal of breast tissue, and the prediction was defeated by the actions that followed it.

Whether their effects are self-fulfilling or self-defeating, however, reflexive predictions have received the kind of attention they have precisely because of their ability to change things for better or worse. If, in the case of SFPs, the mere making of a prediction can make it so, when not making the prediction would not make it so, how can one be anything but fascinated?

Unfortunately, the focus on self-fulfilling prophecies that have an altering effect on the outcome has misled practitioners as well as scholars about both the mechanism of SFPs and the normative and epistemic issues that they entail.

To remediate, I present an empirically informed theoretical analysis of self-fulfilling prophecies, especially in medicine and biology, that takes into account *all* SFPs—including those that do *not* change the outcome of the prediction. SFPs that alter the outcome, I call *transformative.* Those that do not, I call *operative* [8–10]. The inclusion of operative SFPs as genuine SFPs is crucial. Only in doing so can one understand what is truly problematic about both optimistic and pessimistic predictions that self-fulfill.

While the analysis presented here applies to all kinds of SFPs in practical and automated prediction (including examples of SFPs in education and predictive policing),^1^ I will, for the purpose of this article, lay out the different components focusing solely on the case of medical prognosis, and especially on a case of neuroprognostication. The basic hypothetical scenario is the following:

Consider Chris, an unconscious coma patient in intensive care. Suppose that, according to tests of Chris’s brain activity, Chris is predicted to have a ‘poor outcome,’ which could be death or a prolonged disorder of consciousness like vegetative or minimally conscious state. This prognosis informs treatment decisions about Chris, specifically the decision to withdraw life-sustaining treatment. When life sustaining treatment is withdrawn based on the prediction of ‘poor outcome,’ Chris dies as a result.^2^ This makes the prediction true regardless of whether continued treatment would have led to significant recovery. Thus, the prognosis is a self-fulfilling prophecy.

In what follows, I will first lay out the descriptive account of the SFP and its four conditions (Section “Descriptive account: What are SFPs and how do they work?”) which forms the essential basis for understanding the normative analysis. I will then proceed with the normative analysis (Section “Normative analysis: what is problematic about SFPs?) which illuminates both the epistemic issues SFPs pose as well as their moral relevance. I conclude with a general encouragement on how to use this work for practical application.

## Descriptive account: What are SFPs and how do they work?

In order for a prediction to become self-fulfilling, several conditions must be met. Below, I first list the four required steps for a prediction to be self-fulfilling: credibility, employment, employment sensitivity, and realization (Sects. “Condition 1: Credibility as a prediction”–“Condition 4: Realization of the prediction”). Together they form the basis of the definition of an SFP (Section “Definition: defining the self-fulfilling prophecy”). Finally, I elaborate on the notion of collective SFPs (Section “Expansion: Collective SFPs”).

### Condition 1: Credibility as a prediction

In order to become actionable, a prediction must be relied upon. This means that it must first be given credibility. A prediction can be given credibility for many different reasons.

Suppose a family gives credibility to the prognosis simply because they defer to the medical professional’s trustworthy or authoritative status. Any prediction issued by the professional is then treated with **deferential credibility**, even though the professional may themselves grant credibility to the prognosis for a variety of reasons.

For example, in other work [14] I describe an instance of predictive infrastructure where the monitor reveals to the practitioners the predicted outcome, circumventing the need for the medical professional to understand the underlying patterns that led to the prediction. The practitioner can thus grant **default credibility** to the prediction which informs, without further scrutiny, the next step of processing or behavior. Especially with automated prognostic tools, depending on how they are designed and implemented, default credibility may be the norm.

In contrast, when professionals analyze the patterns themselves manually, they nevertheless rely on a theory or model that is already treated as credible. For instance, if they rely on the predictive model that an iso-electric pattern is predictive of a poor outcome, the prognosis is given **theoretical credibility**.

Family members and practitioners may give credibility to a prediction of poor outcome, even if they believe that there remains a degree of uncertainty, simply out of fear for the undesirable consequences that such outcome can entail. **Fear-induced credibility** can be granted for the sake of forestalling these consequences.

In contrast, consider an instance where the prognosis predicts full neurological recovery. It may be that this prediction is treated as credible, regardless of remaining uncertainty, simply because it is agreeable. **Agreeable credibility** can be given due to a combination of moderate plausibility and something akin to wishful thinking.

As will be shown later, collective SFPs typically are subject to **herd credibility**. This occurs when a prediction is given credibility because others give it credibility.

Placebo-effect is a typical example of a prediction that can be given **employment dependent credibility**. There is an expectation that predicting (here, the efficacy of treatment) and acting upon that prediction will have a self-fulfilling effect. For that reason alone, the prediction can be given credibility.

The examples listed here comprise a non-exhaustive list of sources of credibility. Credibility can be granted for other reasons. Furthermore, it is common for several sources of credibility to coincide. A family’s granting credibility to a prognosis may be the result of a combination of deferential, theoretical, fear-induced, and herd credibility. Granting credibility to the prediction that a sugar-pill may have a curing effect [5], can be a combination of theoretical, agreeable, and employment-dependent credibility.

This variety in potential sources of credibility distinguishes practical prediction from prediction in its main scientific role. In prognostic research, for instance, a prediction is granted credibility simply when it is entailed by a theory that is under consideration. An example is the theory that cEEG measuring brain activity patterns over time can project how much brain damage a patient has suffered [15–18]. The credibility granted here is a warrant for a narrow range of further actions that are part of research and development. Namely, observing whether the outcome was as predicted in order to further confirm or disconfirm the theory.

When analyzing the credibility given to predictions, it is important to note that mere assertions, when facing varying degrees of uncertainty, can be given credibility as a prediction too. Therefore, my account requires a broad notion of ‘prediction.’ The essence is that a degree of uncertainty is met with a degree of assertoric force. Predictions, like assertions—but unlike suppositions, questions, speculations, etc.—are intended to allow the hearer or recipient of the prediction to rely upon it as true, or at least actionable [19]. Thus, I define a prediction as *a record (a momentary or persistent information bearing state) of a proposition about a future, or otherwise uncertain observation, or other unknown state of affairs, which is presented with something akin to assertoric force*.

To make this more tangible, consider the example of normative statements regarding what physiological outcomes constitute a ‘poor’ or ‘good’ outcome. Such statements are assertions that implicitly hold within them a degree of uncertainty. In this case, the uncertainty is due to potentially varying values. In other words, such statements are subject to normative uncertainty, i.e., uncertainty about whether the (foreseen) physiological effects of the treatment constitute a desirable outcome [8]. Consider the assertion ‘severe neurological damage constitutes a good outcome.’ This is a signal, intended to be informative, about a proposition that hitherto had been regarded as less certain.

While uncertainty in medical statements is often implied, it is equally often transparently expressed. Prognostic assertions, for example, can make explicit mention of chance or probability. This does not diminish the assertoric force but rather strengthens it. Especially when a factual claim or assertion about the likelihood of an outcome is reinforced by mentioning evidence or results of analysis such as test results, probability calibration, or statistical models, this will increase the likelihood of credibility. In fact, the rhetoric in medicine and science in general, *invites* credibility. A persuasive example is: ‘Clinical studies show that nearly 90% of patients with out-of-hospital cardiac arrest do not survive until discharge.’

Generally, the greater the assertion, the more powerful the prediction. A diagnosis, for instance, is still subject to uncertainty, given that diagnoses can be wrong (e.g. in cases of false positives or false negatives). Yet, a diagnosis may be considered more certain than a prognosis on the basis that it is about a given state rather than a future state, and therefore be given with greater assertoric force. Precisely due to the degree of assertion, the diagnosis is likely to be given further credibility as a prediction, more so than a prognosis given with less assertion even if the latter is understood as a genuine prediction, more so than the former.

As such, these informative signals can be given credibility as a prediction, when the receiver—even the sender—is unaware of or uncareful about the uncertainty that is really at stake. This means that no one may in fact realize that a prediction was made. Receivers of information may simply think they were given facts or neutral claims. Whether or not an assertion like a diagnosis or a normative statement is recognized as a prediction, if it is given credibility as a prediction, it becomes actionable as a prediction.

When studying any given case for potential self-fulfillment, practitioners and researchers can use these insights and examples to analyze whether and how the credibility condition is met in that particular case. Furthermore, one may identify which types of credibility are legitimate, whether there are some that are not, design ways to diminish faulty credibility, and thereby reduce the risk of undesirable self-fulfillment.

### Condition 2: Employment of the prediction

Credibility makes a prediction eligible for employment but giving credibility to a prediction does not guarantee that that prediction will be used. Credibility as a condition is thus insufficient. As soon as the prediction is relied upon, however, one can say that the prediction is employed.

There are many explicit ways in which a prediction can be employed. For instance, once the intensivist relies upon the prognostic test, that prognosis is used even if the prediction is not employed to inform treatment decisions on whether to withdraw life-sustaining treatment. A physician can use prognostic data to inform other treatment decisions without ever communicating the information itself. Or, more likely, a physician can employ the prediction merely by informing the patient’s family about what outcome they can expect. The family, in turn, can rely on the physician’s assertion about the outcome of the patient. The family may employ the prediction to consider the wishes of the patient and inform the physician. Finally, the prediction can be employed to inform treatment, including decisions on withdrawal of life-sustaining treatment.

The employment of a prediction thus consists of the actions and operations performed *based on* the prediction. Typically, as in the example of a prognostic test (e.g. for breast cancer, for brain damage after cardiac arrest), these actions or operations constitute the immediate, if not the ultimate, purpose for which the prediction was produced.

While explicit linguistic expression of the prediction is one paradigmatic example of the ways in which predictions are typically employed, it does not always have to be an oral declaration such as the doctor informing the family. Since linguistic expression can also happen through display on a screen or publishing in some other way, these expressions can happen automatically. As I illustrate elsewhere [14], one result of such automation is that medical professionals—and sometimes family—can see the prognostic information before the neurologist officially informs them.

Perhaps the most common way for humans to employ a prediction is to form an expectation of, and often an opinion about, the predicted outcome. When a prognosis is communicated and given credibility, it automatically yields an expectation of that outcome. That expectation typically comes with a value judgment or, at the very least, questions on how to value the prediction. In standard human contexts, these simple employments of the prediction thus follow immediately on the heels of credibility. In some cases, credibility may even be equated to a type of subconscious employment, as with the expectation-confirmation mechanism [6]. Cognitively treating a prediction as credible, immediately yields a belief of the prediction and an expectation of that realization.

The computational equivalent is simply storing the predicted value for subsequent reference which usually also entails some way of categorization. Take, for instance, the automated generation of a predictive value through continuous electroencephalogram monitoring (cEEG), which is simultaneously categorized as good, poor, or uncertain [14]. Value judgements and categorizations are typical employments where input has been used for further inference or processing. These are relatively simple deductive inferences. More complex processing, like predictive modeling, where the content of the present prediction serves as an input for another prediction task are common in medical prognosis. In another piece [9], I elaborate on how exactly these processes can lead to and exacerbate self-fulfilling prophecies, explaining their moral implications.

Employment of the prediction is absolutely essential. Without it, a prediction is inert and can never be self-fulfilling. If one thinks of credibility as opening a door, employment is pushing the prediction across the threshold.

### Condition 3: Employment sensitivity

On the other side of the threshold lies the object the prediction is about. This object can be sensitive to the employment of prediction. SFPs are necessarily employment sensitive. To put it in the most basic terms, in order to self-fulfill, the object of prediction must somehow be sensitive to the prediction. Only when it is sensitive to the prediction’s employment can the prediction itself have an impact. For that impact to be self-fulfilling, the object of prediction must have either *interpretation sensitivity* leading to interpretative fulfillment or *substance sensitivity* leading to substantive fulfillment. I will demonstrate the difference between the two sources of fulfillment by using examples of the placebo effect.

Consider a patient who has been suffering from acid reflux, chest pain, and shortness of breath. Assume that the prediction ‘this pill will make the patient better’ is given credibility by the patient even if the medicine in question is just a sugar pill which has no inherent medicinal properties. The patient employs the prediction by taking the pill. Consecutively, the patient starts feeling better. Their symptoms disappear completely. This is a typical scenario of placebo-effect in medicine observed in most clinical trials. Now consider two variations of this scenario.

First, assume that the patient’s symptoms were initially diagnosed to be the result of heartburn caused by a hiatal hernia, meaning the upper part of the patient’s stomach bulges through their diaphragm into their chest cavity [20]. In one scenario, after the patient takes the placebo pill and their symptoms disappear, the patient’s diagnosis is checked again and the hiatal hernia can no longer be detected. This would mean that the fulfillment was substantive. The actual substance the prediction was about transformed to match the prediction. In this particular case, it is difficult to explain what causes the substance sensitivity because it is not always clear what causes a hiatal hernia. It may be that the promise of getting better reduced the patient’s stress to the point where they physically relaxed enough to reduce the persistent and intense pressure on the surrounding muscles that were causing the hernia. Whatever the mechanism,^3^ the substantive self-fulfillment of the placebo is due to **substance sensitivity**.

Now consider an alternative scenario in which a second diagnosis, post-placebo, reveals that the hiatal hernia is still present. Moreover, further examination shows that the hernia still causes acid reflux. Yet, the patient no longer experiences chest pain or shortness of breath and does not experience the acid reflux as cumbersome as the way they used to. The patient’s being ‘better’ here is due to interpretative fulfillment. It is the interpretation of the substance the prediction was about, not the substance itself, that made the patient better. This fulfillment is thus due to **interpretation sensitivity**.

As this example shows, whether interpretation sensitivity can be the source of fulfillment depends on how the prediction is formulated. Specifically, it depends on whether the formulated object of prediction leaves room for interpretation. If the original assertion had been ‘this pill will *cure* the patient,’ would that prediction still have fulfilled itself? Much depends on what one considers ‘curing.’ If curing entails the patient’s lack of experienced symptoms, then the answer is yes because the ways in which the patient experiences symptoms is an observer-dependent property. 

In contrast, one may expect that the examination of the patient’s physical state and the diagnosis of hiatal hernia consists of objective measures that remain identical over time. Hence, both the evaluations before and after the placebo was administered are subject to the same standards, leaving little room for subjective interpretation. As such, these measures are not as observer-dependent. Ultimately, the more specific the description of a prediction is, the smaller the room for interpretation will be. If the prediction is ‘taking this sugar pill will reduce the patient’s stress, thereby restoring the natural position of their stomach, fixing the hiatal hernia, and subsequently, make all symptoms disappear,’ the room for interpretation will be significantly reduced, if not eradicated. This, however, does not prevent an SFP from occurring. The prediction could still be self-fulfilling due to substance-sensitivity.

Finally, it is preferable to speak of a **system** rather than an object of prediction being sensitive to employment. This way one can encompass a broad range of interactions between the prediction(s) and the predicted, and the diverse factors that may mediate those interactions. In the section on Collective SFPs below, I demonstrate how the assertion that specific physiological outcomes are either good or poor can become self-fulfilling due to either interpretative or substantive fulfillment. It will become increasingly clear that SFPs do not always unfold by the prediction’s employment operating directly upon the objects or characteristics mentioned in the prediction. An assertion that severe neurological damage entails a poor outcome may affect the valuation of that physiological outcome in a roundabout, indirect way [8, 21].^4^ Focusing on systems allows us to acknowledge a wide range of mediating factors and indirect effects.

To analyze whether a case is subject to potential self-fulfillment, it is necessary to first identify each and every instance of employment and then check whether the system could be sensitive to any of them.

### Condition 4: Realization of the prediction

Regardless of the source of fulfillment, whether SFPs are fulfilled due to substance or interpretation sensitivity, the actual realization of the SFP can be operative or transformative. As I stated at the beginning of this article, there is a fundamental misconception that SFPs are relevant only when substantive or interpretative fulfillment impacts the actual outcome so that it turns out different from what the outcome would have been, had no prediction been made.

To understand this, recall Chris’ case, where a poor prognosis led to the withdrawal of life sustaining treatment. This employment of the prediction directly caused Chris’ death whether or not Chris would have died otherwise. To see the significance of this, compare the following two hypothetical cases.

Imagine Avery and Blake. Like with Chris, their prognosis indicates a poor outcome. Based on those predictions, life sustaining treatment is withdrawn, in effect leading them to pass away. For both of them, it was the prediction and its employment that led to their immediate deaths. Assume, however, that in Avery’s case, the prediction did not change the outcome. The prognosis was based on valid information (a true positive) and Avery would have died anyway. Here, the employment did not change the outcome, but it did affect the *way* the outcome came about. For Avery, the employment of the prediction was operative in bringing about the outcome, even if the same outcome would have been realized without the prediction and its employment. This is an ***operative self-fulfilling prophecy***.

Now, assume that the prognosis Blake received was based on an incorrect understanding of the patient’s condition (a false positive). Blake would have actually survived without any severe disability had life-sustaining treatment been continued. Now the SFP did not only change the mechanism of death; it *caused* Blake’s death who would have otherwise lived. It flipped the outcome from good to poor. In such a case, the predicted outcome is not merely explained by the prediction; the predicted outcome *depends* on the prediction. An SFP that flips the outcome to match the prediction, I call ***a transformative self-fulfilling prophecy***.

Clearly, transformative SFPs demand immediate concern in ways that operative ones do not, as the former has immediate *ethical* implications. However, when operative SFPs are not recognized as true SFPs, significant *epistemic* implications emerge. Not recognizing an operative SFP as a genuine SFP is the same as not acknowledging that the genuine cause of a boxer’s death during a match is the accidental lethal blow of his opponent, just because—counterfactually—the boxer would have collapsed from heart failure soon thereafter. Failing to recognize this is an epistemic failure. As I will show in the second part of the article, recognizing both transformative and operative SFPs as genuine SFPs is epistemically and, consequentially, ethically important because, in practice, it is not self-evident how to distinguish between the two. In fact, it may be impossible to find out, in retrospect, whether the SFP did or did not change the outcome. In neuroprognosis, not having complete certitude about the eventual fate of the patient is precisely the problem. Finding out, in retrospect, what *could have* been is impossible. As I will explain in the normative analysis below (Section “Current concerns regarding unintended outcomes and accountability”), this inability to distinguish between operative and transformative SFPs lies at the heart of the problems that SFPs pose.

Finally, it is important to note that predictions, especially optimistic ones, can be given credibility and be employed, yet while the object of prediction is—at times—sensitive to the employment, the employment does not end up realizing the prediction because the employment sensitivity requirement in that particular instance failed. Because they do not self-fulfill, they are not SFPs. As I will show (in Section “Taking practical and epistemic responsibility for self-fulfilling prophecies”), these predictions are nevertheless relevant to the normative analysis, as they further blur the line between the different types of SFP and no SFP at all.

### Definition: defining the self-fulfilling prophecy

The 4 steps discussed in the previous sections form the conditions that are individually necessary and jointly sufficient for a prediction^5^ to be self-fulfilling:

#1 Credibility as a prediction - The prediction is treated as credible;

#2 Employment of the prediction - Some action or operation constitutes the employment of the prediction;

#3 Employment sensitivity - The system the prediction is about, in its substance or interpretation, is sensitive to the employment of the prediction;

#4 Actual realization - The prediction is realized, either operatively or transformatively, because the object of prediction is sensitive to and affected by the prediction’s employment.

In light of the account explaining the mechanism of an SFP, I define an SFP as follows:

*A self-fulfilling prophecy is a prediction, treated as credible enough to be employed, and realized due to the subject of prediction being situated in a system that is sensitive to and affected by the way the prediction has been employed.*^6^

While this definition of SFP is significantly different from the original one by Merton who coined the term in 1948 [23], it offers several meaningful contributions required to understand the normative problems regarding SFPs.

First, Merton’s definition “The self-fulfilling prophecy is, in the beginning, a false definition of the situation evoking a new behavior which makes the originally false conception come true” [23, p. 195] applies only to transformative SFPs and is therefore incomplete. Furthermore, excluding operative SFPs in that way hides an important characteristic of SFPs. Namely, that it is difficult, and sometimes impossible, to distinguish between transformative and operative SFPs. This means that the limitations of Merton’s account would apply not only to transformative SFPs alone but, additionally, only to those that can be *recognized* as transformative, or potentially transformative. This is unacceptable. As I will show, these kinds of epistemic problems are precisely at the heart of the normative relevance of SFPs that has either been missed or called attention to in the past.

Second, this account diverges from Wilkinson’s, even though Wilkinson does in fact acknowledge many of the epistemic challenges that SFPs pose [11, 12]. This is because Wilkinson, unlike Merton, implicitly acknowledges the difficulty of distinguishing between operative and transformative SFPs, but precisely because of this difficulty, lumps the two together, calling an SFP in intensive care a “self-reinforcing prophecy” [11, 12]. However, this label is epistemically unhelpful in that it does not allow conceptual differentiation between ‘operative’ and ‘transformative’ self-fulfillment. Furthermore, ‘reinforcing’ may be epistemically misleading since, in either case, it is the employment of the prediction that actually explains the outcome predicted. In a transformative SFP especially, the prediction and its employment are altogether forcing, not reinforcing, the outcome. Finally, even with an operative SFP creating the same outcome, ‘reinforcement’ does not signal any change of mechanism, despite the fact that, in operative cases, the would-be means by which the outcome would be realized is displaced, not reinforced. In an operative SFP, the prediction and its employment become operative, *in place of* the mechanism by which the prediction would have been realized.

I argue that the account and definition of the SFP given here corrects mistakes of the past, thereby making it possible to either highlight the moral relevance that was previously missed or clarify epistemic issues that have been identified and called attention to but have been poorly understood. In what follows, I offer a normative analysis of the practical and epistemic issues SFPs pose and how one fails to take responsibility for them. Before I do this, I expand the descriptive account with a short elaboration on collective SFPs.

### Expansion: Collective SFPs

This account, so far, describes individual instances of SFP. Yet, there are also cases where many predictions, or many employments of the same prediction, lead to self-fulfillment. The famous, non-medical example is a bank run after public announcement that the banks will crash. Such collective self-fulfilling prophecies are at times very relevant to medical research as well as clinical practice. In light of this, I now offer a modicum on collections of employments that are together self-fulfilling**.**

One of the most common examples of collective SFPs in medicine, although they have not yet been recognized as such, relates to the medicalization of specific ‘conditions’ and their public evaluation. Typical examples are conditions outside the norm like deafness, dwarfism, Down syndrome and neurodivergence. Medicalization is the perception and treatment of these conditions as health defects or disabilities, often implying that they require fixing or eradication^7^ [25, 26].

When a specific condition is asserted as being a disability that requires treatment, and that assertion is given credibility, it is much more likely that treatment will be offered and accepted. As such, the treatment of the condition equals the employment of the prediction. The effect of many such predictions receiving credibility and subsequently being employed, is that an increasingly smaller population of people with this condition remains. Unfortunately, this may also create troublesome effects of further alienation of, and pressure to assimilate, for those that remain. As such, medicalization has been understood as a form of social control [26, 27]. Moreover, and most relevant to this discussion, the effects of a reduced population can further reinforce the assertion that the condition is indeed a disability that needs fixing and eradicating, thereby fulfilling the original prediction for those who had not given it credibility before.

The sources of self-fulfillment in these scenarios can be interpretative as well as substantive depending on what happens as a result of the population reduction. Take, for instance, in the case of deafness, treatment by way of cochlear implants. By systematically ‘fixing’ deafness, its prevalence is reduced. Perhaps that is the right course of action. I do not mean to make a value judgment here. However, the confrontation and experience of the majority (e.g. the hearing population) with the condition (e.g. deafness) is increasingly limited and, as a result, the minority (e.g. the deaf community) is increasingly misunderstood. While deafness, to those who live with it, is in no way more or less of a disability than before the prediction was made, the hearing community increasingly interprets it as one. This is *interpretative fulfillment*. However, majority rule tends to also have substantive effects on society, its structure, its organization, and its services. When a reduced population leads to a reduction in schools, reduced transmission of sign language, or general exclusion, deafness will, in effect, become more debilitating. The employment of the majority’s prediction can thus create an environment in which the minority is increasingly experiencing their condition as a limitation rather than a mere trait. This is *substantive fulfillment*.^8^

Whether interpretative or substantive, collective SFPs thus require a collection of employments. In such cases, the prediction, once given credibility, can prompt the decision to treat a single occurrence of deafness with a cochlear implant. But no single instance of such employment of the prediction is sufficient for self-fulfillment. The system is only sensitive to the combined effect of many such employments. These employments can be the result of a singular prediction, given credibility by many, or multiple predictions given individual credibility but predicting the same outcome. Either way, it is the collection of employments that are together self-fulfilling.

What is fascinating about these types of collective SFPs, is that they work in both directions and can thus often be reversed, if so desired [8]. In many societies, for instance, sign language use is mostly limited to deaf people. Typically, hearing people in such societies perceive being deaf as a serious problem requiring professional intervention [28]. This often leads to self-fulfilling feedback such as in the example above. In contrast, societies like those on Bali and Martha’s Vineyard, have adopted widespread use of sign language because of a substantial deaf population. As a result, being deaf is regarded as a trait there, not a disability [29]. This example provides an idea of how such collective SFPs can be reversed. Imagine a population asserts that deafness is a trait, perhaps to some unfamiliar yet a valuable trait nonetheless, and as a result sign language is taught in schools for the hearing. Suppose it is offered alongside other foreign languages and students could just pick among them. This would give self-fulfilling feedback in the other direction, asserting that deafness is rather exotic but not something that requires fixing. Furthermore, there would be substantive realization in the other direction too because, with the majority mastering sign language, the deaf population would not be as hindered in their day-to-day life, effectively being relieved from previous inability, or supposed disability.

Although I highly encourage further study of collective SFPs, I limit my account here to what is needed for illuminating some instances of self-fulfillment of normative claims or value judgments.^9^ I now turn to the normative analysis of self-fulfilling prophecies, based on the descriptive account given above.

## Normative analysis: What is problematic about SFPs?

Now that SFPs have been clearly described, it is possible to better analyze what is problematic about them. The descriptive account has serious implications for the ways in which one must take practical and epistemic responsibility for the consequences of SFPs. I will first discuss the existing concern (Section “Current concerns regarding unintended outcomes and accountability”.) and then correct the epistemic critique I find inadequate (Section “Taking practical and epistemic responsibility for self-fulfilling prophecies”).

### Current concerns regarding unintended outcomes and accountability

The first reason that makes distinguishing between operative and transformative SFPs so important is that the focus of practitioners as well as critics has almost exclusively been about the risk of transformative SFPs^10^ (e.g. in the neuroprognostication case [11–13, 30–33]). It is easy to see why. As explained at the beginning of this article, they have much more immediate practical consequences that require one’s attention. Clearly, the difference between causing an inevitable death (as with an operative SFP) or one that could have been prevented (as with a transformative one) is significant.

It is therefore no surprise that the focus on transformative SFPs, especially with pessimistic ones, has also led to a concern that SFPs help evade responsibility in an important way, regarding the practical consequences of the outcome. If looking only at instances where the prediction was not only responsible for the outcome but the outcome *depended* on the prediction and would not have been realized otherwise, where does that leave us with regard to accountability? Clearly the predictor, or those who gave credibility to or employed the prediction, are responsible for bringing about the outcome. But are they also accountable?

The existing concern is that SFPs evade accountability because of some epistemic failing. The epistemic failing, intuitively, seems to be about some unjustified belief about what brought about the realization of the prediction. If one gives credibility to a positive test result, assuming it is a true positive when in fact it is a false positive, one is underestimating the uncertainty that the prediction is subject to. However, the unjustified belief can no longer be about giving credibility to the prediction, even on false grounds, since the prediction ends up being accurate. This is not a question of accuracy. The fallacy then is a mistaken belief about what (or who) makes the prediction accurate [34, 35]. Crucially, it is about being unaware of the role one played in bringing about the outcome. As Biggs puts it, “The actors within the process—or at least some of them—fail to understand how their own belief has helped to construct that reality” [34, p. 295].

The argument regarding responsibility that follows is that *if* there is total unawareness, then one cannot be answerable for producing the outcome and therefore one cannot be held accountable. And thus SFPs, by cloaking the agents’ contribution, help evade accountability for those who help bring about the outcome. I argue, however, that there are some serious problems with this argument.

First of all, unaccountability due to unawareness in the past does not entail unaccountability for future instances. I would argue that if there is total unawareness and if one cannot be blamed for that unawareness, then one can indeed not be held accountable. However, unawareness is only an excuse for unaccountability as long as one is not to blame for the unawareness. This means that, even if one was not responsible for a mistake in the past, once there is awareness about the mistake, one may be held accountable for reproducing the mistake in the future. Clearly, if the problem is a lack of awareness, then the solution is an increase of awareness. I will return to the importance of increased awareness later since it may not be significant here, as my next point will prove.

Second, unawareness may not be as relevant an argument as one thinks it is when it comes to practical prediction. In medical prognosis, early-diagnostics, and clinical research, for example, there often is no unawareness, as practitioners may be well aware of the potential consequences and even intend them. Let us look at cases where there is explicit acknowledgment of the potential for transformative SFPs and their consequences.

For those that have a positive effect, like with placebo, the awareness will obviously not give reason to prevent employment of the prediction.^11^ On the contrary, if the prediction self-fulfills, great! In fact, the prediction is employed *in order* to self-fulfill*.* For optimistic predictions, awareness will thus not lead to non-employment. But even explicit acknowledgment of pessimistic SFPs potentially being transformative may not prevent an agent from employing the prediction. Take patients in coma for whom transformative SFPs result in a poor outcome when a poor outcome could have been prevented. Once aware of this possibility, different practitioners make different decisions while taking this risk into account. Based on the acknowledged uncertainty, some practitioners may indeed choose to avoid employment of the prediction altogether [30]. They may choose not to withdraw life-sustaining treatment. But this may come at a significant cost to the patient as well as to the distribution of resources to other patients. To avoid such negative consequences, some physicians may choose to err on the side of withdrawing treatment anyway.

In essence, because clinical practice is subject to so much uncertainty, whether the prediction is employed and how, is often based on a mixture of time-sensitivity, balancing costs and benefits and, ultimately, it is a judgment call. In medicine, unawareness is often not the problem and awareness of the risk does not necessarily entail non-employment of the prediction. Even if the practitioner is responsible, they may still just be doing the best they can, precisely because they are aware of how much uncertainty there is. Of course, uncertainty is inherent to prediction. Without uncertainty, there would be no need to predict.

Medical decision-making in general, and intensive care decisions especially, are marred by uncertainty. For instance, think about an unconscious patient who likely has condition X. I say ‘likely’ because the patient shows symptoms of X and the intensivist, based on their experience, can recognize it as a potential case of X. Yet, when a scan is made to test the presence of X, it does not show up on the scan. This, as intensivists confirm, does not mean that X is not there, it just means they cannot find or prove it. Now imagine that, to the best of the intensivist’s knowledge, treatment Y is likely to improve the patient’s condition. As a result, the intensivist predicts that the patient will benefit from treatment Y. However, there is a small chance that the patient is allergic to treatment Y. If the patient is indeed allergic to treatment Y, this would cause massive complications in their condition. Now, the intensivist can test the patient for allergies but receiving test results would require a wait of three hours and the patient will most benefit from the treatment if they get it as soon as possible and at the very latest within the next hour.

To be clear, this is a regular prediction, or asserted educated guess, (not necessarily self-fulfilling) made in light of uncertainties. The intensivist is fully aware of the risk they are taking by administering the treatment. Is the intensivist responsible for the complications when it turns out the patient is indeed allergic to the treatment? In some sense, sure. But holding them accountable for the consequences of their decision seems beside the point. In practical prediction, predictions generally take place in situations where decision and action are desired, despite deficient knowledge of all the relevant factors. Being aware about the effect of one’s actions does not solve the problem. Therefore, unawareness is not the issue. When uncertainty faces time pressure, cost and benefits must be weighed and a judgment call must be made, often prematurely. There simply may be no better way of responding. Administering the treatment may be the most responsible thing to do, even if it later turns out that it was a mistake.

When it comes to SFPs, this last bit will prove to be crucial: ‘if it later turns out that it was a mistake.’ I will now correct for what I believe to be an inadequate epistemic analysis of SFPs.

### Taking practical and epistemic responsibility for self-fulfilling prophecies

The big difference between other, regular predictions and self-fulfilling prophecies is that with SFPs, if a similar error was made, and the initial judgment call was flawed, it will not later *turn out* that it was a mistake because the SFP will prove the prediction right even if the initial judgment call was flawed.

Typically, error signals indicate when a mistake was made. This makes the receiver of such error signal epistemically fortunate as they are now in a position to learn and improve. Both an error signal and the lack of an error signal are informative. Ideally, a lack of error signals can then reliably be interpreted as that the correct decision was made. Not so with self-fulfillment. By never producing error signals, the feedback produced by SFPs is always positive and can never be relied upon.

Think of the hypothetical cases of Avery and Blake, discussed above (Section “Condition 4: Realization of the prediction”.). Blake would have supposedly survived had life sustaining treatment been continued, hence making it a transformative SFP. But how would anyone know? In real life, once the prediction is employed by ways of withdrawal of life-sustaining treatment, the patient dies and it cannot be found out in retrospect what would have happened instead.^12^ Unlike in the case of treatment Y, where a physician can observe complications and allergic responses as clear error signals, Blake’s death produces no such error signals. Even if physicians are aware that it may have been a mistake, they do not find out whether it really was a mistake. What’s worse, when Avery passes away after withdrawal of life-sustaining treatment, even if the judgment call there was correct and Avery would have passed away anyhow, the intensivist cannot find out that the judgment call was right either. The lack of error signals here is not a reliable sign of positive feedback. As such, the receiver of feedback following an SFP will be epistemically unfortunate.

SFPs, because they entail their own fulfillment, do not produce reliable feedback. And if one cannot accurately distinguish failure from success, one cannot accurately learn from past decisions either. This is the central epistemic failing of SFPs.

It is now evident why it is very difficult, in practice, to differentiate between operative and transformative SFPs, or between true or false test results. Knowing the difference between an operative and a transformative SFP depends on knowing what would have happened instead, if no prediction had taken place, and knowledge about such counterfactuals is difficult. Not knowing when the prediction changed the outcome and when it did not is precisely why no lesson can be learned.

While this is equally true for optimistic as well as pessimistic SFPs, it may seem most urgent that attention is paid to learning about the pessimistic ones because their practical consequences are dramatic while those of optimistic SFPs are desirable. Yet, when it comes to epistemic failing, optimistic SFPs may be even harder to learn from, simply because the employments are likely to be much more complex and, consequentially, the employment sensitivity may be intractable.

I mentioned above (in Section “Condition 4: Realization of the prediction”) the normative importance of predictions that are potentially subject to self-fulfillment due to the system—at times—being sensitive to the employment but end up not self-fulfilling because the employment sensitivity requirement in that particular instance failed. It is important here to distinguish between employments of optimistic and those of pessimistic predictions.

Consider a patient in coma with a poor prognosis whose ventilator is withdrawn at a time where patients are not expected to be able to breathe without the ventilator’s help. The ventilator is removed, motivated by the expectation that the patient will pass away soon thereafter but the patient nevertheless starts breathing on their own. Unexpectedly, the system was not sensitive to the employment of the prediction. The prediction does not self-fulfill, the patient’s survival is an error signal.

While employment of a pessimistic prediction typically leads to limiting options and thereby reducing possibilities, employment of optimistic predictions tends to increase options, thereby increasing possibilities. With optimistic predictions, the system, even if potentially sensitive, is given much more room to respond to the prediction’s employment in a variety of ways.

Let me illustrate this with the placebo effect. The prediction ‘this pill will make the patient better’ when employed by communicating it to the patient and subsequently by administering a sugar pill, can have a transformative self-fulfilling effect making the patient better when they would not have recovered otherwise. The employment can also have an operative effect, making the patient better even if they would have gotten better anyway. Finally, the employment can have no effect at all, thus not making it an SFP. Either the patient does not get better, in which case one receives an error signal, or the employment can have no effect at all but will still appear to be self-fulfilling because the patient got better anyway for other reasons unknown [1, 3]. In this last case, even though the prediction does not self-fulfill, it appears as though it did.^13^

As mentioned, the inability to distinguish between operative and transformative SFPs lies at the heart of the epistemic problems that SFPs pose. Optimistic predictions that end up not self-fulfilling (but appear as though they do^14^) muddy the waters further by making it hard to assess whether a transformative SFP, an operative SFP, or no SFP at all was at work. Optimistic self-fulfilling prophecies are thus even harder to clearly identify as such.

If the epistemic problem with SFPs is that they inhibit learning, where does that leave us regarding responsibility? The general concern that ‘SFPs evade accountability in an important way due to an epistemic failing’ still rings true. The epistemic failing being, however, that SFPs inhibit learning. Therefore, the problem with SFPs is not that they help evade responsibility for the practical outcomes they create. Rather, they allow for agents to evade responsibility about their failure to learn. Perhaps some might think this is not a great moral injustice. After all, is there an obligation to learn?^15^

First of all, agents are likely to learn anyhow. Unconscious cognitive processes that automatically incorporate and organize information perceived as feedback lead to all sorts of unconscious—or implicit—learning [40, 41]. When relying on unreliable feedback, because one is unaware of the fact that it is unreliable, this will likely result in flawed learning processes. Specifically, one can learn to reproduce mistakes as though they were correct. Second, in medicine, there is at least one area where practitioners are expected to learn correctly—and that is in research and development. Especially in prognosis, because there is so much uncertainty, researchers are constantly seeking to improve the ability to accurately predict. How is one supposed to improve a practice when mistakes and successes cannot be recognized as such?^16^

I argue that correct learning is the most essential requirement for innovation. Responsible innovation therefore requires a critical approach to one’s own learning processes. One must know **when** one is learning, **what** one is learning, and **how** one is learning.

Here, finally, awareness is of crucial importance. If an agent is unaware of how SFPs are skewing their learning process *and* the agent is not to blame for that unawareness, then they cannot be held accountable. But, as I stated before, not being blameworthy for mistakes in the past does not entail being unblameworthy for future instances.

The relevance of increased awareness about one’s failure to learn is not unlike that of increased awareness with regard to implicit bias. Agents may have unconscious cognitive processes that result in “automatic associations, of which [they] may not be aware, that are difficult to control, and may conflict with [their] professed beliefs” [44, p. 1]. It may be difficult to hold an agent accountable for the unintended, practical consequences of their judgments and actions caused by such implicit biases. These agents “may, however, be blamed if they fail to act properly on the knowledge that they are likely to be biased e.g. by investigating and implementing remedies to deal with their biases” [45, p. 24]. I argue that, similarly, agents can be held accountable if they fail to act properly on the knowledge that they are likely to err in their learning processes.

Improving one’s ability to learn will require in depth study of the relationship between the employment of predictions and the sensitivity of the systems the predictions are about. Distinguishing between optimistic and pessimistic predictions will prove to be crucial in that regard. While delving into this in further detail is outside the scope of this article, it is important to note that the employments of optimistic versus pessimistic predictions differ greatly in how they affect the range of employment sensitivity at hand. For now, it suffices to say that, in the case of self-fulfilling predictions in medicine, pessimistic SFPs are ethically more problematic in that missing an instance of transformative SFP always has ethical ramifications. In contrast, the epistemic issues with optimistic SFPs do not lead to ethical issues outside of epistemic injustice since transformative cases of SFP are desirable.

## Conclusion

This article offers a descriptive account and normative analysis of self-fulfilling prophecies in medicine to help researchers and practitioners better understand the problems that SFPs entail in medicine and how to address them.

The common concern regarding SFPs is that they transform the predicted outcome to match the prediction. Such *transformative SFPs* indeed have some special moral implications for practical prediction. However, SFPs pose an epistemic challenge as well. To understand the core epistemic challenge, one must recognize and analyze *all* SFPs, including *operative SFPs* that do not change the outcome but only impact *the way in which* the predicted outcome was brought about.

The present descriptive account offers a detailed description of the mechanism of SFPs and their different forms. The four elements; credibility, employment, employment sensitivity, and realization, together form the conditions that are individually necessary and jointly sufficient for a prediction to be self-fulfilling. As such, they form the basis of my definition: ‘a self-fulfilling prophecy is a prediction, treated as credible enough to be employed, and realized due to the subject of prediction being situated in a system that is sensitive to and affected by the way the prediction has been employed.’

The present normative analysis focuses on illuminating SFPs’ core epistemic problem that underlies the ethical ramifications. SFPs inhibit appropriate learning due to their automatic confirmatory feedback, thereby inviting reiteration and exacerbation of mistakes. Whether or not one succeeded or failed to predict the eventual outcome absent of any prediction and its employment, is hard if not impossible to find out. This inability to retrospectively check the validity of the prediction is an impediment for research and innovation. As such, checking for accuracy cannot be a quality assurance strategy whenever there is a risk of self-fulfilling prophecies at work. In medicine especially, where research-practice distinctions are easily blurred, this limitation can have dramatic consequences.

With this work, anyone can now systematically analyze where a given system of prediction is sensitive to employment and investigate what kind of unintended employment(s) may additionally be at play. For practical examples, self-fulfillment has been analyzed based on this account in neuroprognostication [9], outcome measures in fertility treatment [22], and cerebral performance scoring [8]. An analysis of reflexive mechanisms in predictive genetic testing is underway.

As such, using the descriptive account, researchers and practitioners can detect and analyze potential self-fulfilling mechanisms in any given case by checking for each condition whether and how it is met. The normative account can then support an accountable attitude and the responsibility to prevent or at least become aware of the epistemic failure that self-fulfilling prophecies invite. And, as a result, the ethical implications that otherwise follow can be better addressed, remedied, or altogether avoided.

## Footnotes

1 I discuss SFPs in practical and automated prediction more generally in [10]. The descriptive account for SFPs in medicine is in accordance with the general account. However, the normative account is different, and different in significant ways as SFPs in medicine may sometimes be intended (as with placebo) and often lack process mistakes. On the contrary, practitioners typically assume employment sensitivity since the employment is aimed at influencing the predicted outcome [10, p. 143]. Rather than failing to recognize there is employment sensitivity, process mistakes emerge when fundamental mistakes are made about the way(s) in which the employment sensitivity works.

2 This case of SFP has been discussed in bioethics literature [8, 9, 11, 12] and in debates about the quality of medical research [13].

3 Given extra time, the patient may spontaneously recover and do so regardless of the placebo. This could indicate that there was no self-fulfillment at all, though such instance would be hard to distinguish from cases where the placebo was simultaneously effective (see Section “Taking practical and epistemic responsibility for self-fulfilling prophecies”), leading to operative self-fulfillment, as explained below (Section “Condition 4: Realization of the prediction”).

4 For another example of this, see [22].

5 As stated before, this account of prediction includes any situation where a degree of uncertainty is met with a degree of assertoric force.

6 I give simplified versions of this definition in [10]. While slightly more elaborate, this definition makes it relatively easy and straightforward for researchers and practitioners to analyze (the potential of) self-fulfillment in medicine.

7 The Diagnostic and Statistical Manual of Mental Disorders (DSM) infamously used to classify homosexuality and later ‘sexual orientation disorder’ as a mental disorder. Even today, conversion therapy remains an existing practice to attempt to change a person’s sexual orientation, gender identity, or gender expression to align with heterosexual and cisgender norms. [24] The mechanisms of collective SFP apply to this case of gender divergence, as well as to the medicalization of neurodivergence.

8 Another example of this interpretative and substantive fulfillment mechanisms in collective SFP happens with the valuation of Cerebral Performance Categories [8]. Starting from a negative or pessimistic assumption, can then be seen as a vicious cycle [21] which can also be reversed [8].

9 For detailed analysis of other examples, see [8] and [22].

10 The focus on transformative SFPs is such that the term SFP in the literature tends to refer exclusively to transformative ones. Wilkinson could be seen as an exception, since his analysis of the epistemic problems that SFPs pose, led him to propose an alternative label “self-reinforcing prophecy” which implicitly contains both operative and transformative SFPs [11, 12]. I do not adopt this label, for reasons explained above (under Section “Definition: defining the self-fulfilling prophecy”).

11 Here, I refer to the use of placebo for therapeutic purposes, not for the avoidance of placebo in research settings.

12 Attempts of postmortem verification of postanoxic patients exist [36], but they cannot account for the impact of many of the prediction’s employments.

13 An illustrative example of this mistake is one of the most influential publications on placebo “The powerful placebo” [37]. It nearly took half a decade for scholars to show that no assertion could be made that placebo was in fact at work in any of the studies used [3].

14 Pessimistic predictions can in principle be subject to the same problem, but it is much more unlikely as one would need the failure of the employment to go by unnoticed (without producing error signals) and simultaneously have something else, unrelated, happen that realizes the pessimistic prediction.

15 Some scholars in philosophy would argue that yes, one has an obligation to have adequate knowledge of the world. This obligation to ‘know well’ is what philosophers have termed “epistemic responsibility” [38, 39]. I shall leave this debate aside here as there are specific reasons why the obligation to learn, and learn accurately, is important in this context. I lay out the arguments in what follows.

16 Neuroprognostic research especially is known to be subject to self-fulfilling bias and aggravating feedback loops [9, 41, 42].
